# Bcl-x_L_ Blocks a Mitochondrial Inner Membrane Channel and Prevents Ca^2+^ Overload-Mediated Cell Death

**DOI:** 10.1371/journal.pone.0020423

**Published:** 2011-06-02

**Authors:** Daniel Tornero, Inmaculada Posadas, Valentín Ceña

**Affiliations:** 1 Unidad Asociada Neurodeath, Universidad de Castilla-La Mancha, Albacete, Spain; 2 Laboratorio de Inmunobiología Molecular, Hospital General Universitario Gregorio Marañón, Madrid, Spain; 3 Centro de Investigación Biomédica En Red de Enfermedades Neurodegenerativas, Instituto de Salud Carlos III, Madrid, Spain; Case Western Reserve University, United States of America

## Abstract

Apoptosis is an active process that plays a key role in many physiological and pathological conditions. One of the most important organelles involved in apoptosis regulation is the mitochondrion. An increase in intracellular Ca^2+^ is a general mechanism of toxicity in neurons which occurs in response to different noxious stimuli like excitotoxicity and ischemia producing apoptotic and necrotic cell death through mitochondria-dependent mechanisms. The Bcl-2 family of proteins modulate the release of pro-apoptotic factors from the mitochondrial intermembrane space during cell death induction by different stimuli. In this work, we have studied, using single-cell imaging and patch-clamp single channel recording, the mitochondrial mechanisms involved in the neuroprotective effect of Bcl-x_L_ on Ca^2+^ overload-mediated cell death in human neuroblastoma SH-SY5Y cells. We have found that Bcl-x_L_ neuroprotective actions take place at mitochondria where this antiapoptotic protein delays both mitochondrial potential collapse and opening of the permeability transition pore by preventing Ca^2+^-mediated mitochondrial multiple conductance channel opening. Bcl-x_L_ neuroprotective actions were antagonized by the Bcl-x_L_ inhibitor ABT-737 and potentiated by the Ca^2+^ chelator BAPTA-AM. As a consequence, this would prevent free radical production, mitochondrial membrane permeabilization, release from mitochondria of pro-apoptotic molecules, caspase activation and cellular death.

## Introduction

Calcium is one of the most important second messengers in the cells, regulating many pathways that are essential for cell physiology [1] and also for the pathogenesis of different diseases such as Alzheimer's dementia [2], brain ischemia or epilepsy, where Ca^2+^ signaling plays a pivotal role [3].

Increased intracellular Ca^2+^ levels triggers apoptosis or programmed cell death in different cell types [4]–[6]. The function of Ca^2+^ in apoptosis is a complex subject involving the interplay between many systems, such as redox systems, the stress-activated protein kinase cascade and the Ca^2+^ signaling pathway [7].

The mitochondria play a key role regulating the apoptotic mechanisms and also regulate some forms of necrotic cell death [8], [9]. Calcium overload induces mitochondrial inner membrane permeabilization (MIMP) that promotes mitochondrial swelling, outer membrane rupture and release of intermembrane proapoptotic proteins such as cytochrome C and apoptosis inducing factor (AIF) to the cytoplasm [10]. These proteins also activate caspases and, subsequently, caspase-activated DNase [10].

Evidence supporting mitochondrial Ca^2+^ accumulation after excitotoxic stimulation comes from data on the effects of mitochondrial inhibitors on free cytosolic Ca^2+^ [11], changes in mitochondria membrane potential [12], [13] and elevation of free mitochondrial Ca^2+^ [14]. The key role of mitochondrial Ca^2+^ accumulation in delayed excitotoxic cell death is suggested by the fact that respiratory uncoupler-induced depolarization of mitochondrial membrane potential (Δψm) during glutamate exposure is neuroprotective [15]. In addition, it has been shown that the binding of Ca^2+^ to cardiolipin, a phospholipid present in the mitocondrial inner membrane, can convert the adenine nucleotide translocase (ANT) into a large unselective channel that would be responsible for the activity of the multiple conductance channel (MCC), which is able to cause MIMP [16].

The intrinsic apoptotic pathway is mainly regulated by proteins that belong to the Bcl-2 family through their actions on the mitochondria and also through their ability to hetero- or homodimerize with other proteins of the same family [17]-[19]. Mitochondrial events associated with apoptosis induction and the effects of differential expression and activity of members of the Bcl-2 family of proteins have been reported to occur in response to Ca^2+^ overload, for example in the ischemia-reperfusion damage [20]–[22]. In addition, it has been described that, even under non-apoptotic conditions, some members of the Bcl-2 family of proteins modulate gene expression [23] and that some members of this family might also regulate intracellular Ca^2+^ homeostasis [24], [25], suppressing Ca^2+−^induced mitochondrial MCC activation [26] and controlling, in this way, the release of pro-apoptotic factors from mitochondria. Moreover, Bcl-xL prevents 6-OHDA induced-death and blocks mitochondrial multiple conductance channel activation [27].

In this work, we have studied, using single-cell imaging and patch-clamp single channel recording, the mechanism involved in the protective effect of Bcl-xL on ionomycin-mediated Ca^2+^ overload in human neuroblastoma SH-SY5Y cells. We have found that Bcl-xL neuroprotective actions take place at mitochondria where the protein delays both mitochondrial potential collapse and opening of permeability transition pore by preventing Ca^2+−^mediated mitochondrial MCC opening. This prevents apoptotic cascade activation and cellular death.

## Results

### Bcl-x_L_ protects SH-SY5Y cells against ionomycin-induced toxicity

We have studied the effect of Bcl-xL over expression on ionomycin-induced death in the human neuroblastoma cell line SH-SY5Y. Cell cultures were stably transfected with either DNA containing the open reading frame of Bcl-xL subcloned into pcDNA3 (SH-SY5Y/Bcl-xL) or with empty/pcDNA3 (SH-SY5Y/Neo). The SH-SY5Y/Bcl-xL cell line has been thoroughly characterised [28] and is resistant to staurosporine-mediated cell death [29]. Bcl-xL expression in these cell lines was analysed by western blot. As previously described [27], Bcl-xL levels, measured in total cellular extracts, were significantly higher in SH-SY5Y/Bcl-xL cells than in the SH-SY5Y/Neo cells. Over expressed Bcl-xL was found to be highly expressed in abundant in SH-SY5Y/Bcl-xL over SH-SY5Y/Neo cells (Supporting information Fig. S1).

**Figure 1:**
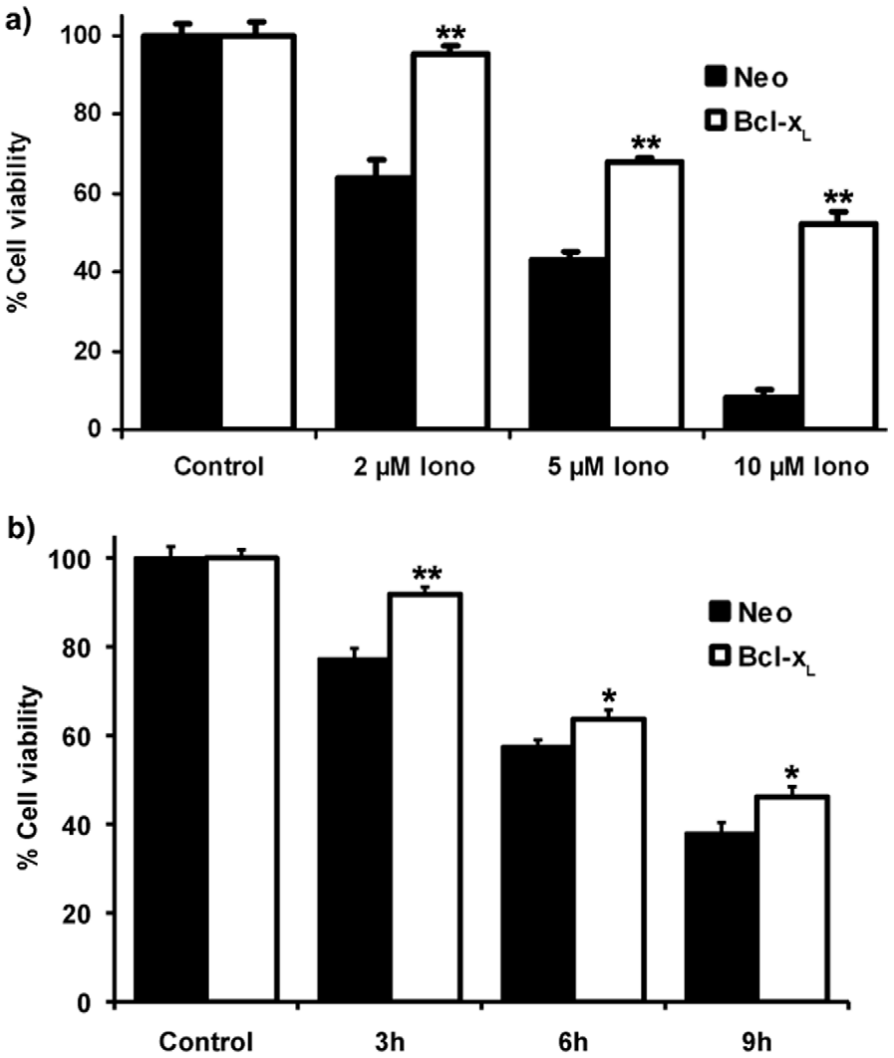
Effect of Bcl-x_L_ over expression on ionomycin-induced death in SH-SY5Y cells. a) Cell viability of SH-SY5Y/Neo (black bars) and SH-SY5Y/Bcl-x_L_ (white bars) cells treated with ionomycin for 3 hours at different concentrations. Afterwards, cell viability was determined using the MTT assay. Data represent mean ± S.E.M. of 3 experiments carried on by quadruplicate. ***p<0.001 as compared to control cells. b) Time-course of cell viability, determined by MTT assay, of SH-SY5Y/Neo (black bars) and SH-SY5Y/Bcl-x_L_ (white bars) cells exposed to ionomycin (2 µM). Data represent mean ± S.E.M. of 3 experiments carried on by quadruplicate. **p<0.01, ***p<0.001 as compared to control cells.

**Figure 2:**
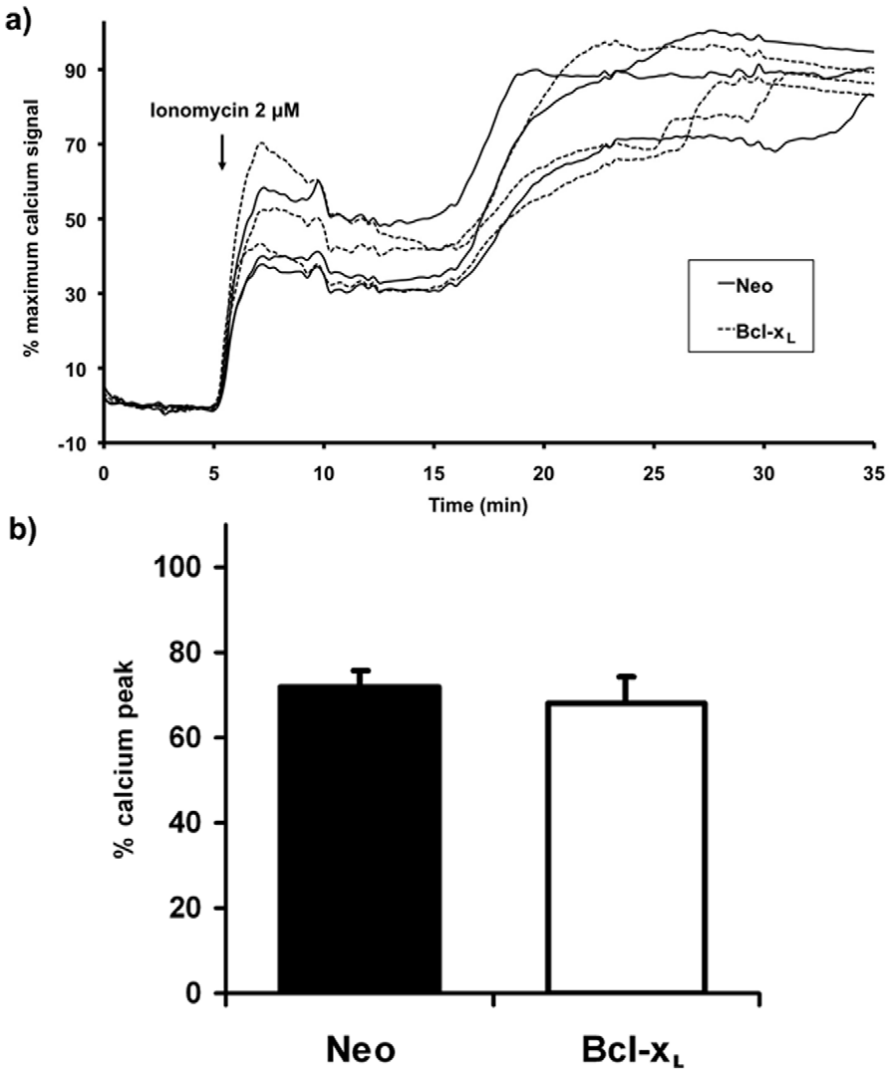
Lack of effect of Bcl-x_L_ over expression on ionomycin-mediated increase in Ca^2+^ levels. a) Time-course of Ca^2+^ levels, expressed as percentage of maximum Ca^2+^ response, following ionomycin (2 µM; arrow) addition to SH-SY5Y/Neo (solid lines) and SH-SY5Y/Bcl-x_L_ cells (dashed lines). Following an initial Ca^2+^ peak, a late Ca^2+^ increase occurs reaching a plateau that is taken as the maximum Ca^2+^ level for the neuron. b) Cytoplasmatic response to ionomycin treatment (2 µM) in SH-SY5Y/Neo (n = 10) and SH-SY5Y/Bcl-x_L_ (n = 11) cells measured as the ratio of the early Ca^2+^ peak to the maximal ionomycin signal obtained during each individual experiment. Data represent mean ± S.E.M of the indicated number of experiments.

We used the MTT cell viability assay method to analyze the effects of ionomycin-mediated Ca^2+^ overload on SH-SY5Y cells. As it can be observed in Fig. 1, ionomycin induced a marked dose-dependent decrease in cell viability in control cells after 3 h of treatment. Bcl-xL over expression protected SH-SY5Y cultures against ionomycin (2 µM) (Fig. 1) without affecting ionomycin-mediated increase in Ca^2+^ levels (Fig. 2). Treatment of SH-SY5Y/Neo cells with ionomycin resulted in morphological changes typical of apoptosis, including cell shrinkage, rounding and detachment from the plate, as observed under phase contrast microscopy (data not shown).

### Caspase activity

**Figure 3:**
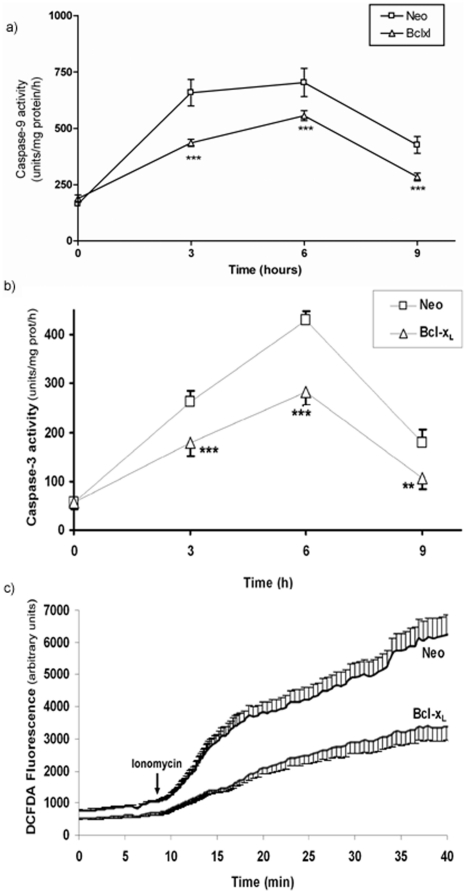
Caspases activation and free radical production. a) Time-course of ionomycin-induced caspase-9 activation. Caspase-9 activity in SH-SY5Y/Neo and SH-SY5Y/Bcl-x_L_ cells treated with ionomycin (2 µM) for 3, 6 and 9 hours was determined. Results are expressed in units of fluorescence/mg of protein/h and represent mean ± S.E.M. of 3 experiments. ***p<0.001 as compared to 0 h time. b) Time-course of ionomycin-induced caspase-3 activation. Caspase-3 activity in SH-SY5Y/Neo and SH-SY5Y/Bcl-x_L_ cells treated with ionomycin (2 µM) for 3, 6 and 9 hours was determined. Results are expressed in units of fluorescence/mg of protein/h and represent mean ± S.E.M. of 3 experiments **p<0.01, ***p<0.001 as compared to 0 h time. c) Time-course of ionomycin-induced increase in free radical production. Ionomycin (2 µM; arrow) was added to both SH-SY5Y/Neo or SH-SY5Y/Bcl-xL cells and free radical production was measured on-line as an increase in DCFDA fluorescence. Each data point represents mean ± S.E.M. of 15 individual cells.

Ionomycin (2 µM) induced a time-dependent increase in both caspase 9 and caspase-3 activity that reached a maximum peak of activity at 3 h for caspase 9 (Fig 3 a) and at 6 h for caspase 3 activity (Fig. 3b) after ionomycin addition. However, when the experiment was performed using cells that over express the anti-apoptotic protein Bcl-xL, a significant reduction in ionomycin-mediated caspase 9 (Fig. 3a) and 3 (Fig. 3b) activity was observed. Moreover, the ionophore also produced an increase in free radical production in neuroblastoma SH-SY5Y cells measured by an increase in DCFDA fluorescence (Fig. 3c) suggesting that free radical production is involved in ionomycin-mediated neuroblastoma cell death. Bcl-xL over expression partially blocked the increase in ionomycin-mediated free radical production.

### Bcl-x_L_ over expression prevents Ca^2+^-induced MCC opening

**Figure 4:**
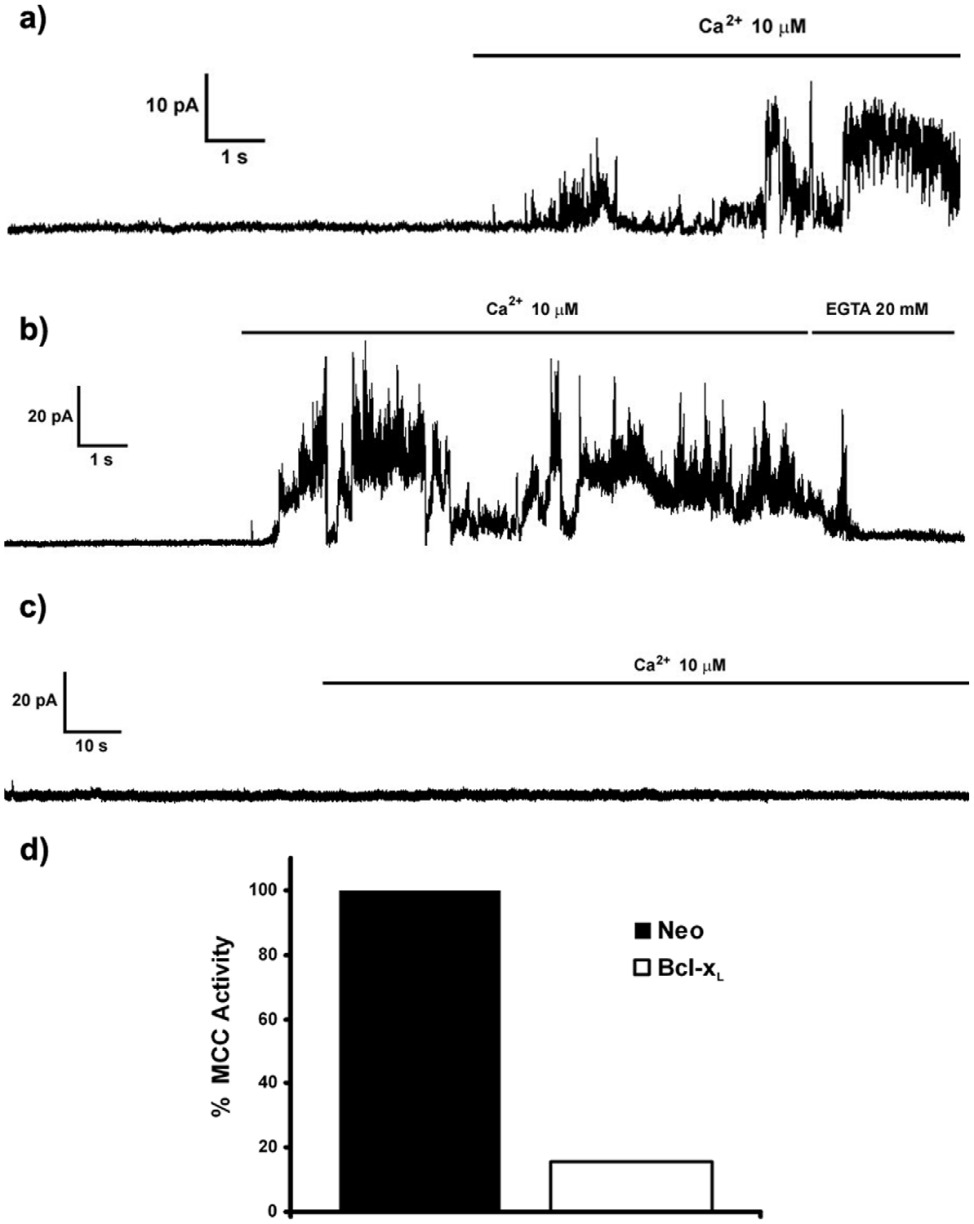
Bcl-x_L_ blocks the Ca^2+^-induced MCC activation in isolated mitoplasts. Mitoplasts were prepared and single channel activity was recorded as indicated in Materials and Methods. a) Induction of MCC activity following increase in Ca^2+^ concentration (upper bar) in SH-SY5Y/Neo cells. b) Inhibition of Ca^2+^-induced MCC activation by Ca^2+^ chelation in SH-SY5Y/Neo. EGTA 20 mM was added after Ca^2+^ stimulation (upper bars). c) Same protocol as in a) performed in mitoplasts obtained from SH-SY5Y/Bcl-x_L_ cells. No channel activity can be observed after 10 µM Ca^2+^ addition despite the channel was present in the patch (supplemental material figure S1). d) Percentage of patches where a rise in Ca^2+^ (10 µM) induced MCC activity provided that the channel was present in the patch. This was tested at the end of the experiment as indicated in Material and Methods. Data represent the percentage of patches where the channel was opened for SH-SY5Y/Neo (n = 10) and SH-SY5Y/Bcl-x_L_ (n = 13) cells.

One of the proposed models responsible for the initiation of apoptosis is the opening of a MCC in the mitochondrial inner membrane [30], that allows the release of cytochrome C (cyt C) leading to activation of the intrinsic apoptotic pathway. The activity of MCC was studied using single channel recording in SH-SY5Y mitoplasts. Typical MCC recordings in mitoplasts from SH-SY5Y/Neo and SH-SY5Y/Bcl-xL over expressing cells were indistinguishable when recordings were performed at negative pipette potentials (−60 mV) due to the high open probability that the channel shows at this potential (data not shown). This property was used to test the presence of the MCC in the patch at the end of the experiments (Supporting information Figure S2). Neither the basal activity nor the voltage-dependence of the channel activation was modified in mitochondria isolated from Bcl-xL over expressing cells (data not shown). However, at a +30 mV pipette potential and a Ca^2+^ concentration of one nM, the open probability is low and MCC remained mainly at the closed state. At this pipette potential, the increase in Ca^2+^ concentration from one nM to 10 µM, induced an increase in MCC activity in SH-SY5Y/Neo mitoplasts in every patch assayed where MCC was present (n = 10) indicating channel activation (Fig. 4a). This activation was quickly reversed by Ca^2+^ chelation using EGTA (Fig. 4b). However, in SH-SY5Y/Bcl-xL isolated mitoplasts, 10 µM of Ca^2+^ only induced MCC opening in two out of the 13 seals where MCC was present (Fig. 4c, 4d), suggesting that Bcl-xL over expression prevents Ca^2+^-induced MCC opening.

### Bcl-x_L_ over expression inhibits the decrease in ΔΨ_m_ induced by ionomycin

**Figure 5:**
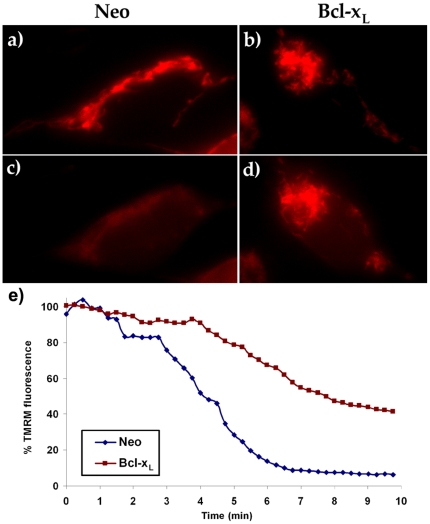
Bcl-x_L_ prevents ionomycin-induced lost in ΔΨ_m_. TMRM fluorescence in SH-SY5Y/Neo (a and c) and SH-SY5Y/Bcl-x_L_ (b and d) cells exposed to 2 µM ionomycin at time 0 h. a) and b) time  = 0; c) and d) time  = 5 min. e) Time-course of TMRM fluorescence in mitochondria-rich cytoplasm versus the nucleus that is considered background in SH-SY5Y/Neo (red trace) and SH-SY5Y/Bcl-x_L_ (blue trace) cells. The complete videos for these experiments can be found in Supporting information Video S1 and Video S2. This experiment was repeated 4 times with similar results.

Ionomycin (2 µM) induced a rapid lost in Δψm in SH-SY5Y/Neo cell line that was almost complete 6 minutes after the drug application (Figs. 5a and c and Supporting information Video S1). However, over expression of Bcl-xL in these cells markedly delayed this process (Figs. 5b and d and Supporting information Video S2). Five minutes after ionomycin addition, TMRM fluorescence in the mitochondrial-rich cytoplasmic region was decreased to about 30% of the maximum value in SHSY5Y/Neo cell line while it remained at around 80% of maximum value in SH-SY5Y/Bcl-xL cells (Fig. 5e).

### Bcl-x_L_ over expression decreases the MIMP induced by ionomycin

**Figure 6:**
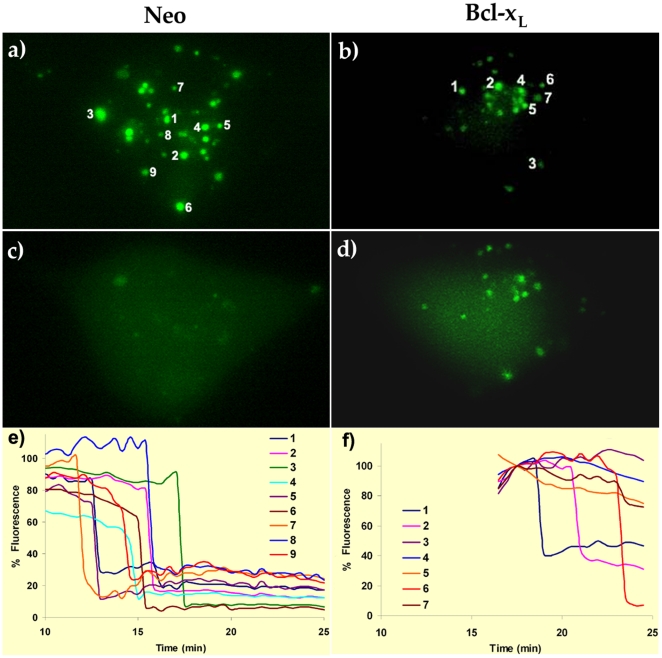
Bcl-x_L_ decreases ionomycin-induced MIMP. SH-SY5Y cells were loaded with calcein as indicated in Material and Methods. Calcein fluorescence in SH-SY5Y/Neo (a and c) and SH-SY5Y/Bcl-x_L_ (b y d) cells exposed to 2 µM ionomycin at time 0 h. a) and b) time  = 0; c) and d) time  = 20 min. Numbers represent individual mitochondrion whose time-course is represented in panels e) and f). Time-course of fluorescent changes in individual mitochondrion from e) SH-SY5Y/Neo and f) SH-SY5Y/Bcl-x_L_. The complete videos for these experiments can be found in Supporting information Video S3 and Video S4.

Ionomycin (2 µM) induced MIMP opening in SH-SY5Y cells as followed by calcein release from mitochondria. As it can be observed in figures 6a and 6c and Supporting information Video S3, 10 minutes after the addition of the Ca^2+^ ionophore, the calcein staining began to disappear from the mitochondria in the SH-SY5Y/Neo cells and 15 minutes after the ionophore application, all mitochondria have lost the calcein fluorescence (Fig. 6e) suggesting MIMP opening.

However, Bcl-xL over expressing SH-SY5Y cells delayed ionomycin-induced calcein release from mitochondria (Fig 6b and d and Supporting information Video S4). Moreover, 25 minutes after the ionomycin addition only few mitochondria have lost the calcein staining (Fig 6f).

### Effect of BAPTA-AM and ABT-737 on ionomycin-mediated toxicity on SH-SY5Y cells

**Figure 7:**
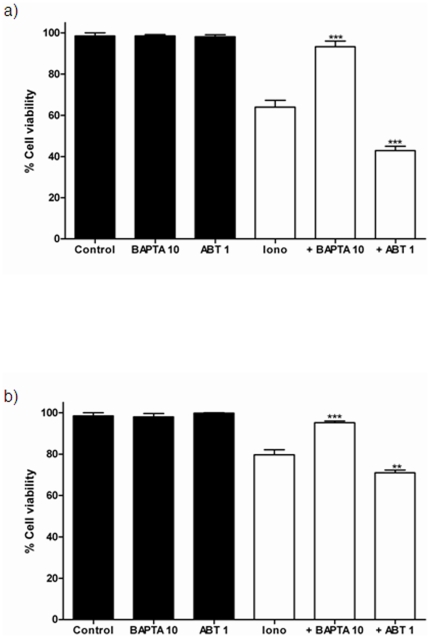
Effect of BAPTA-AM and ABT-737 on ionomycin-induced toxicity in SH-SY5Y cells. The effect of BAPTA-AM and ABT-737 on ionomycin-induced toxicity was studied in both a) SH-SY5Y/Neo and b) SH-SY5Y/Bcl-x_L_ cells treated with either vehicle, BAPTA-AM (10 µM; BAPTA 10) or ABT-737 (1 µM; AB 1) for 30 minutes and then either Ionomycin 2 µM (Iono) was added in the absence or presence of BAPTA-AM 10 µM (+BAPTA 10) or ABT-737 1 µM (+ABT 1) for 3 hours and cell viability was determined using the MTT assay. Data represent mean ± S.E.M. of 12 individual cells from 3 different experiments. **p<0.01, ***p<0.001 as compared to Ionomycin-treated cells.

To gain insight into the cause-effect relationship between ionomycin-mediated Ca^2+^ overload and the observed effects on SH-SY5Y, we studied the effect of ionomycin in the presence of a Ca^2+^ chelator like BAPTA-AM and in the presence of the Bcl-xL inhibitor ABT-737 [31], [32]. As it can be observed in Fig. 7a, The presence of the Ca^2+^ chelator BAPTA-AM (10 µM) markedly decreased ionomycin-mediated toxicity in SH-SY5Y/Neo cells. Moreover, the presence of the Bcl-xL inhibitor AB-737 (1 µM) significantly potentiated ionomycin-mediated toxicity (Fig 7a). In Bcl-xL over expressing SH-SY5Y cells, BAPTA-AM was also able to partially prevent ionomycin-mediated toxicity (Fig. 7b) suggesting that Ca^2+^ was the ion responsible for it. Morever, the Bcl-xL inhibitor ABT-737 has a slight potentiating effect of ionomycin-mediated toxicity (Fig 7b). This effect was small, probably due to the high level of expression of the Bcl-xL protein achieved in these cells (Supporting information Fig. S1).

### Effect of BAPTA-AM and ABT-737 on ionomycin-mediated effects on ΔΨ_m_ and MIMP

**Figure 8:**
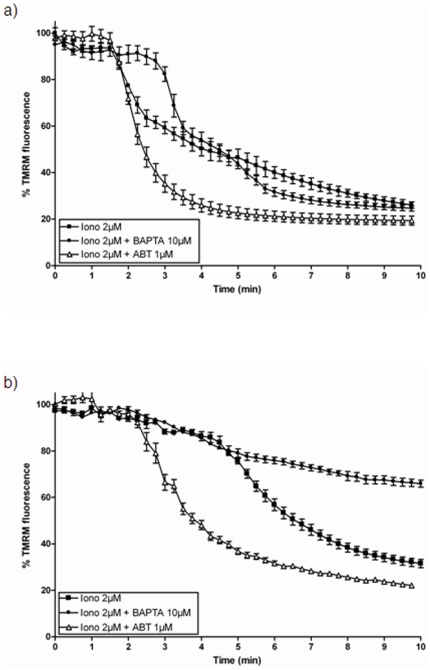
Effect of BAPTA-AM and ABT-737 on ionomycin-induced Δψ_m_. The effect of BAPTA-AM and ABT-737 on ionomycin-induced Δψ_m_ was studied in both a) SH-SY5Y/Neo and b) SH-SY5Y/Bcl-x_L_ cells treated with either vehicle, BAPTA-AM (10 µM) or ABT-737 (1 µM) for 30 minutes and then loaded with TMRM. Afterwards, cells were washed in Krebs solution and Ionomycin (2 µM; Iono) was added and Δψ_m_ was measured on-line as a decrease in TMRM fluorescence. Each data point represents mean ± S.E.M. of 15 individual cells from 3 different experiments.

**Figure 9:**
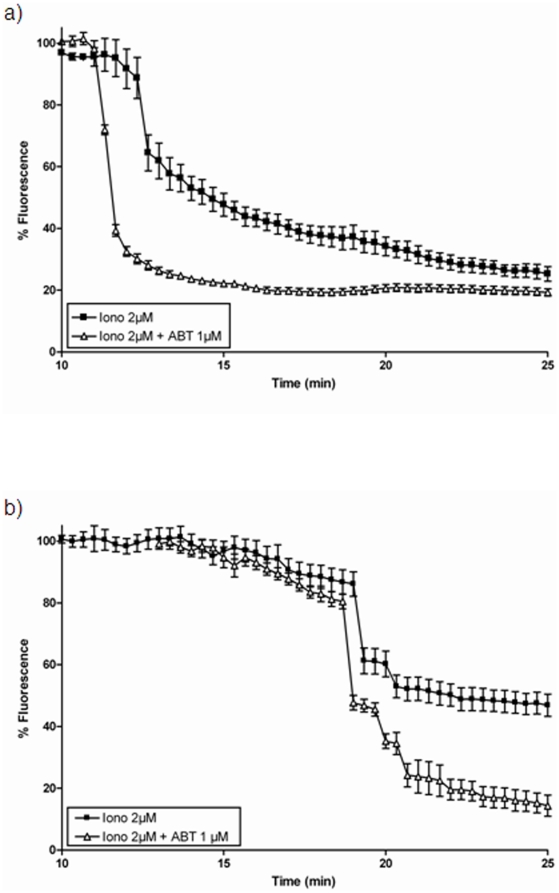
Effect of ABT-737 on ionomycin-induced MIMP. The effect of ABT-737 on ionomycin-induced MIMP was studied in both a) SH-SY5Y/Neo and b) SH-SY5Y/Bcl-x_L_ cells treated with either vehicle or ABT-737 (1 µM) for 30 minutes and then loaded with calcein as indicated in Material and Methods. Afterwards, cells were washed in Krebs solution and Ionomycin (2 µM; Iono) was added and MIMP was measured on-line as a decrease in mitochondrial calcein fluorescence. Each data point represents mean ± S.E.M. of 15 individual cells from 3 different experiments.

To explore the actions of Bcl-xL at the mitochondrial level following an ionomycin-mediated Ca2+ overload, we exposed SH-SY5Y cells to ionomycin in the presence of either the Ca2+ chelator BAPTA-AM (10 µM) or of the Bcl-xL inhibitor ABT-737 (1 µM). As it can be observed in Fig. 8a, the presence of BAPTA-AM delayed the beginning of Δψm collapse in the SH-SY5Y/Neo cells while ABT-737 induced a faster Δψm collapse suggesting that Bcl-2 familiy members might play a physiological role counteracting the Δψm collapse induced by Ca2+ overload during different insults. When the same experiment was repeated in Bcl-xL over expressing SH-SY5Y cells, BAPTA-AM completely prevented the Δψm collapse and only a slow, but sustained depolarisation was observed (Fig. 8a) suggesting that the combination of lower free Ca2+ levels due to ion chelation by BAPTA-AM and Bcl-xL over expression have a strong protective effect against ionomycin-mediated Δψm. Moreover, in the presence of ABT-737, ionomycin-induced a much faster Δψm collapse (Fig. 8b). Moreover, ABT-737 caused an increase in the rate of MIMP induced by ionomycin in SH-SY5Y/Neo cells (Fig. 9a) while the effect in Bcl-xL over expressing SH-SY5Y cells was much smaller (fig. 9b), probably due to the high level of Bcl-xL expression in these cells (Supporting information Fig. S1). The effect of BAPTA-AM on MIMP could not be studied because BAPTA also chelates Co2+ that is required to quench extramitochondrial calcein allowing visualization of the calcein-labelled mitochondria and so the study of MIMP.

### Effect of BAPTA-AM and ABT-737 on ionomycin-mediated caspase activity

**Figure 10:**
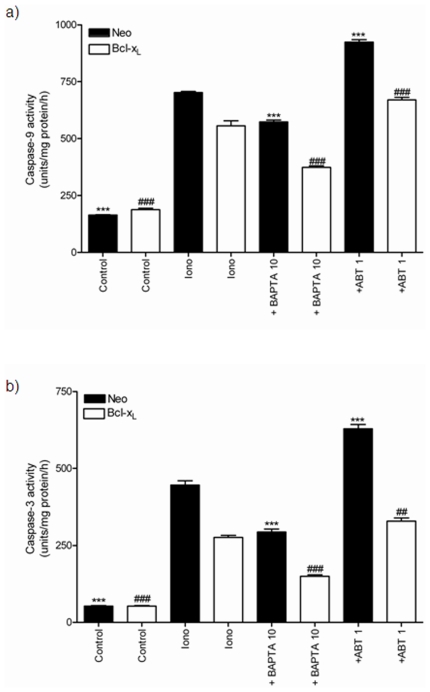
Effect of BAPTA-AM and ABT-737 on ionomycin-induced caspasas activation. a) Caspase-9 activity measured in total lysates obtained from SH-SY5Y/Neo (black bars) and SH-SY5Y/Bcl-x_L_ (white bars) cells treated with vehicle or Ionomycin (2 µM; Iono) in the presence or absence of BAPTA-AM 10 µM (+BAPTA 10) or ABT-737 1 µM (+ABT 1) for 6 hours. Data represent mean ±S.E.M. of 12 experiments. ***p<0.001 as compared to Ionomycin-treated SH-SY5Y/Neo cells. ^###^p<0.001 as compared to Ionomycin-treated SH-SY5Y/ Bcl-x_L_ cells. b) Caspase-3 activity measured in total lysates obtained from SH-SY5Y/Neo (black bars) and SH-SY5Y/Bcl-x_L_ (white bars) cells treated with vehicle or Ionomycin (2 µM; Iono) in the presence or absence of BAPTA-AM 10 µM (+BAPTA 10) or ABT-737 1 µM (+ABT 1) for 6 hours. Data represent mean ± S.E.M. of 12 experiments. ***p<0.001 as compared to Ionomycin-treated SH-SY5Y/Neo cells. ^##^p<0.01, ^###^p<0.001 as compared to Ionomycin-treated SH-SY5Y/ Bcl-x_L_ cells.

Consistent with the observed effects of Ca2+ chelation by BAPTA-AM and Bcl-xL inhibition by ABT-737 on Δψm and MIMP, BAPTA reduced ionomycin-mediated increase in caspase 9 and caspase 3 activity in both SH-SY5Y/Neo cells and Bcl-xL over expressing SH-SY5Y cells, while ABT-737 potentiated it (Fig 10). This would suggest that Bcl-xL would play a physiological role counteracting the deleterious effects of Ca2+ overload.

## Discussion

Cellular Ca^2+^ overload is a common fact in several pathological situations including ischemia and neurodegenerative diseases [33], [34]. Calcium accumulation in the mitochondrial matrix produces a dysfunction in this organelle mediated by reactive oxygen species (ROS) and causes the release of proapoptotic factors resulting in cell death [35], [36]. Here, we present direct evidence showing that Ca^2+^-overload leads to activation of mitochondrial MCC, collapse of mitochondrial potential, mitochondrial internal membrane permeabilization, caspase 3 activation and cell death. The antiapoptotic protein Bcl-xL decreases Ca^2+^-overload-induced neuroblastoma cell death acting downstream of Ca^2+^ entry, likely, by blocking Ca^2+^-mediated opening of the mitochondrial MCC and delaying both Ca^2+^-mediated Δψm and MIMP. This leads to a lower caspase 9 and caspase 3 activation and to a decrease in neuronal death.

Calcium might convert the ANT transporter into a large unselective channel, through its binding to cardiolipin [36]. This modulates the activity of MCC that is responsible for the mitochondrial internal membrane permeabilization [16]. The electrophysiological experiments presented here show that the observed channel has the same characteristics of reversibility and voltage-dependence as previously reported for the MCC (Supporting information Figure S1) [16]. Bcl-xL does not interfere with ionomycin-mediated Ca^2+^ entry, but efficiently prevents Ca^2+^ activation of the mitochondrial MCC, interfering with the initial step in the signalling pathway that leads to cell death.

Following ionomycin-treatment, there is a rapid lost in Δψm that precedes an increase in the permeability of the mitochondrial inner membrane that would lead to cytochrome c release and activation of effector caspases like caspase 3. Bcl-xL does not modify ionomycin-mediated increase in intracellular Ca^2+^ levels or voltage-dependence of the MCC channel. However, it blocks Ca^2+^-mediated activation of mitochondrial MCC and, subsequently, ionomycin-induced decrease in Δψm and MIMP. This effect is not synchronic in all mitochondria, because functional heterogeneity of mitochondria with respect to mitochondrial redox state, membrane potential, respiratory activity, uncoupling proteins and mitochondrial Ca^2+^ determines different responses to many signals and may vary their sensitivity to different noxious stimuli resulting in different time courses in the individual mitochondrial responses (Supporting information Video S1 and Video S3). This suggests the relevance of studying the participation of these organelles in apoptotic processes at the individual cell or mitochondrion level [17]. The fact that the Bcl-xL inhibitor ABT-737 [31], [32] antagonised the Bcl-xL protective actions strongly suggests that Bcl-xL plays a key role in the cell defense against Ca^2+^ overload-mediated toxicity. Moreover, ABT-737 also potentiates ionomycin-mediated toxicity in SH-SY5Y/Neo cells suggesting that the Bcl-2 family members might be participating in the physiological response to a Ca^2+^ overload insult.

Although, it has been described that Bcl-xL might act on different mechanisms involved in apoptosis regulation [38], including inhibition of Apaf-1-dependent caspase 9 activation [39], and that Ca^2+^ influx can activate calpain producing Bid cleavage, which targets the mitochondria causing apoptosis [40], the most plausible explanation for the antiapoptotic action presented here is a direct effect of Bcl-xL on the mitochondrial MCC. Previous studies have shown that Bcl-xL, despite its preferent location in the outer membrane, can also be found at the inner membrane [41] and there is ample evidence of direct interaction of the anti-apoptotic proteins of the Bcl-2 family with ANT, via its BH-4 domain [42], [43]. This Bcl-xL action on the mitochondrial MCC, downstream of Ca^2+^ entry, would act on the MIMP preventing the lost of the Δψm and the subsequent release of apoptotic factors from the intermembrane space. This would block caspase 3 activation and cell death. The fact that the Ca^2+^ chelator BAPTA-AM, potentiated the protective actions of Bcl-xL supports this view, because when less Ca^2+^ ions are available to stimulate the opening of the MCC due to Ca^2+^ chelation by BAPTA-AM, the protective actions of Bcl-xL would be more intense. However, contribution of a Ca^2+^-mediated increase in mitochondrial ROS to Ca^2+^-mediated cell death cannot be excluded [44]. Blockade of this action by Bcl-xL might also contribute to its protective action on Ca^2+^ overload-mediated cell death.

In summary, calcium overload, as occurs in different pathological situations, increases mitochondrial Ca^2+^ that, on one hand, opens the mitochondrial MCC leading to MIMP that would collapse the mitochondrial potential causing MOMP that would release cyt c, activate caspase and produce apoptotic death. Bcl-xL might block this toxic cascade by preventing Ca^2+^-mediated mitochondrial MCC opening that would inhibit MIMP and mitochondrial potential collapse.

## Materials and Methods

### Cell culture

SH-SY5Y neuroblastoma cell line was grown in Dulbecco's modified Eagle's medium (DMEM) supplemented with 2 mM L-glutamine, 20 units/ml penicillin, 5 µg/ml streptomycin and 15% heat-inactivated foetal calf serum (Gibco) as reported previously [45]. Cells were maintained at 37°C in a saturated humidity atmosphere containing 95% air and 5% CO2. Constitutively Bcl-xL expressing neuroblastoma SH-SY5Y cells were kindly provided by Dr. Joan Comella [27], [29]. Cells were plated at a density of 4×10^4^ cells/cm^2^ and allowed to grow until 80% confluence was reached.

### Cell viability studies

Cells plated at a density of 4×10^4^ cells/cm^2^ and allowed to grow until 80% confluence were treated with vehicle or Ionomycin at different concentrations for 3 hours, or with vehicle or Ionomycin (2 µM) for different times. In another set of experiments cells were treated with vehicle or Ionomycin (2 µM) in the absence or presence of pharmacological inhibitors (BAPTA-AM 10 µM or ABT-737 1 µM) for 3 hours. After treatments, cell viability was assessed using the 3-[4, 5-dimethylthiazol-2-yl]-2,5-diphenyltetrazolium bromide (MTT) cell survival assays as previously described [45]. Culture medium was removed and 400 µL of MTT (50 µg/mL) in Krebs solution (with the following composition in millimoles/Liter: NaCl 140, KCl 5.9, MgCl2 1.2, HEPES 15, glucose 10, CaCl2 2.5, pH 7.4) were added to each well following ionomycin treatments. This was followed by incubation at 37°C for 30 min. After this, the medium with MTT was removed and 300 µL dimethylsulfoxide (DMSO) were added. Two hundred µL from each well were then transferred to a 96-well microplate and read at 540 nm with a reference wavelength of 630 nm in the dual mode.

### Electrophysiological recording

Cells were resuspended in 10 mL isolation medium (230 mM mannitol, 70 mM sucrose, 5 mM HEPES pH 7.4) and homogenized by hand. Mitochondria were isolated as previously described [27]. Mitoplasts were prepared from mitochondria by passage through a French press at 2000 p.s.i., as previously described [43]. Intact mitochondria were morphologically different from mitoplasts and were easily avoided during patch-clamp experiments. Recording micropipettes (10–40 MΩ) were prepared on a P-97 puller (Sutter Instruments, New York, NY, USA). High resistance seals (>1 GΩ) were obtained by pressing a micropipette onto the membrane of a mitoplast, and applying negative pressure. Patches were excised so that the matrix face of the inner membrane was exposed to the bath. Bath and pipette solutions were identical and contained (in millimoles/Liter): HEPES 5, KCl 150, CaCl2 1, EGTA 55, pH 7.4. Single channel activity was recorded using an EPC-9 patch-clamp amplifier (HEKA, Lambrecht, Germany) and stored on videotape at 5 kHz. Computer analysis of current signals was performed using pClamp 9.0 (Axon Instruments, Union City, CA, USA). Typically, MCC was considered to be present if current transitions showed a conductance >250 pS [46]. The presence of MCC in the patch was tested at the end of the experiment by switching the pipette potential to −60 mV to increase MCC open probability (supporting material Figure S1).

### Fluorescence microscopy studies

SH-SY5Y cells were plated on 20 mm diameter coverslips and allowed to grow until 60% confluence was reached. Fluorescence was observed on an inverted microscope (Nikon Eclipse TE-2000-S, Birlingam, CA, USA) equipped with a 150 W Xenon lamp and 100×, 1.3 numerical aperture, epifluorescence oil immersion objective. Excitation wavelength was selected using a Life Technology monochromator (Omega Optical Inc, Brattleboro, VT, USA). Excitation wavelength was selected using a filter wheel (Sutter “lambda 10”, Novato CA, USA). Images were acquired with a digital camera (ORCA II, Hamamatsu, Shizouka, Japan) and data were analyzed using commercial software (Metamorph, Universal Imaging Corporation, Silicon Valley, CA, USA).

#### Measurement of cytosolic calcium

Fura-2 obtained from Molecular Probes (Invitrogen, Carlsbad, CA, USA) was used at 5 µM and loaded with 0.005% Pluronic in Krebs solution for 20–30 minutes at 37°C in the dark. The coverslips were washed twice with Krebs solution and placed in the fluorescence camera. The dye was excited alternately at 340 and 380 nm allowing ratiometric measurements of changes in cytosolic Ca^2+^ levels. Images were collected at intervals of 15 seconds using an emission filter of 510 nm. When a noxious stimulus is intense, like in the case of ionomycin, a deregulation of Ca^2+^ homeostasis called delayed calcium deregulation (DCD) takes place. This implies a second increase in Ca^2+^ concentration until a plateau that is irreversible. This is named maximum Ca^2+^ signal for that particular cell. To homogenize the data, Fura-2 measurements in every cell are referred to its maximum Ca^2+^ level, which is different for each cell.

#### Mitochondrial membrane potential measurement

SH-SY5Y cells were loaded in 4 nM Tetramehtyl-rhodamine-methyl-ester (TMRM) (Molecular Probes, Carlsbad, CA, USA) in Krebs solution for 20 minutes at 37°C in the dark. In the experiments performed in the presence of pharmacological inhibitors (BAPTA-AM 10 µM or ABT-737 1 µM) cells were pre-incubated with inhibitors for 30 minutes before the incubation with the fluorescent probe. Cells were then washed twice with Krebs solution and fluorescence was observed using an excitation filter of 535 nm and an emission filter of 590 nm. Frames of 10 Z-planes (250 nm thick) were recorded every 15 seconds over 10 minutes to exclude that the switch-off of mitochondrial fluorescence was due to mitochondrial movement away from the focussed microscope plane.

#### MIMP imaging

Calcein-AM penetrates cell membranes and is hydrolyzed by intracellular and intraorganellar esterases, yielding calcein which is hydrophilic and hence membrane-impermeable and trapped within all subcelluar compartments including the mitochondrial matrix [47]. If calcein-labeled cells are then loaded with the divalent cation Ca^2+^, the calcein-dependent fluorescence is quenched in all subcelular compartments with exception of the mitochondrial matrix, because the inner mitochondrial membrane is the only intracellular membrane which is normally Ca^2+^-impermeant. Due to the ability of BAPTA-AM to chelate Ca^2+^ ions, it was not possible to study MIMP in the presence of BAPTA-AM. The selective staining of the mitochondrial matrix disappears upon permeabilization of the inner mitochondrial membrane. While the mitochondrial potential, can be lost and recovered, the loss of calcein fluorescence is irreversible and thus allows for the study of transient openings of inner membrane pores. Calcein imaging was performed as previously described [47]. Briefly, SH-SY5Y cells were loaded with one µM calcein-AM (Molecular Probes) in Krebs containing one mM Ca^2+^ for 30 minutes in the dark. In the experiments performed in the presence of ABT-737 (1 µM), the cells were pre-incubated with the drug for 30 minutes before the incubation with the fluorescent probe. Then, the coverslips were washed twice with Krebs solution and fluorescence was observed using an excitation wavelength of 485 nm and an emission wavelength of 530 nm. Frames of 10 Z-planes were recorded every 20 seconds over 30 minutes to exclude that the switch-off of mitochondrial fluorescence was due to mitochondrial movement away from the focussed microscope plane.

### Reactive oxygen species production

SH-SY5Y cells were loaded in Krebs containing 10 µM 5-(and-6)-chloromethyl-2′,7′-dichlorodihydrofluorescein diacetate, acetyl ester (DCFDA) (Molecular Probes, Carlsbad, CA, USA) in Krebs solution for 20 minutes at 37°C in the dark. In the experiments performed in the presence of pharmacological inhibitors (BAPTA-AM 10 µM or ABT-737 1 **µM**) cells were pre-incubated with inhibitors for 30 minutes before the incubation with fluorescent probe. Cells were then washed twice with Krebs solution and fluorescence was observed using an excitation filter of 535 nm and an emission filter of 635 nm. Frames were recorded every 30 seconds over 40 minutes.

### Caspase activity determination

Caspases-9 and -3 activities were determined as previously described [48]. Cells were grown in 6-well culture plates until 80% confluence was reached. Then, cells were treated with vehicle or ionomycin 2 µM for different times. In another set of experiments cells were treated with vehicle or Ionomycin (2 µM) in the absence or presence of pharmacological inhibitors (BAPTA-AM 10 µM or ABT-737 1 µM) for 6 hours. Afterwards, cells were washed twice with cold PBS and lysed in Lysis buffer containing 100 mM Hepes pH 7.4, 5 mM DTT, 5 mM EGTA, 0.04% Nonidet P-40, and 20% glycerol. Cell extracts were then centrifuged at 5,000 x *g* (10 min, 4°C). For caspase 3 activity cell extracts (40 µg of protein) were incubated in reaction buffer (25 mM Hepes, 10% sucrose, 0.1% CHAPS, 10 mM DTT) containing 50 µM fluorescence sustrate Z-DEVD-AFC at 37°C for 1 h. For caspase 9 activity cell extracts (40 *µ*g of protein) were incubated in reaction buffer (50 mM Hepes, 50 mM NaCl, 0.1% CHAPS, 5% glycerol, 10 mM DTT) containing 50 *µ*M fluorescence sustrate Ac-LEHD-AFC at 37°C for 1 h. Cleavage of the AFC fluorophore was determined in a spectrofluorometer at excitation wavelength of 400 nm and fluorescence was detected at an emission wavelength of 505 nm. Caspase activity was expressed as units of fluorescence/mg of protein/h.

### Western blot analysis

Wester blot analysis was performed as previously described [49]. Briefly, cells were grown in 6-well culture plates until 80% confluence was reached and collected and resuspended in homogenization buffer (10 mM HEPES, 0.32 M sucrose, 100 µM EDTA, 1 mM DTT, 0.1 mM PMSF, 40 µg/ml aprotinine, 20 µg/ml leupeptine; pH 7.4). Cells were homogenated using a polytron (two cycles, 10 s at maximum speed). Homogenates were centrifuged at 3.000x*g* for 5 min and protein content of the supernatants (total lysate) was determined. Samples (30 µg) were loaded on 15% PAGE-SDS and transferred onto nitrocellulose membranes. Membranes were blocked in PBS-Tween 20 (0.1%) containing 5% non-fat dry milk and 0.1 % BSA for 1 h at 4°C and then incubated with polyclonal anti-BCL-xL antibody (1∶1,000) or polyclonal α-tubulin antibody (1∶2,000) overnight at 4°C. Afterwards, blots were washed with PBS-Tween 20 (0.1%) and incubated with HRP-anti-IgG antibody (1∶10,000) for 2 h at 4°C. Immunoreactive bands were visualized using an enhanced chemiluminiscence system (ECL; GE Healthcare, Madrid, Spain).

### Statistical Analysis

Data are expressed as mean ± SEM. Statistical analyses were carried out using the one-way analysis of variance (ANOVA) and the *a posteriori* Bonferroni's *t*-test for multiple comparisons. *P* values less than 0.05 were considered significant. Statistics results are reported in the figure legends.

### Drugs and Chemicals

Z-DEVD-AFC and Ac-LEHD-AFC was from Calbiochem (Madrid, Spain). BAPTA-AM, DCFDA, TMRM, and Calcein-AM were from Molecular Probes Inc. (Barcelona, Spain). ABT-737 was from Selleck Chemicals (Madrid, Spain). Bcl-xL and α-tubulin antibodies were from Cell Signalling (Barcelona, Spain). All other reagents were obtained from Sigma-Aldrich (Madrid, Spain).

## Supporting Information

Figure S1 Bcl-xL expression in SH-SY5Y/Neo and SH-SY5Y/Bcl-xL cell. a) Total lysates obtained from SH-SY5Y/Neo (lanes 1, 2 and 3) and from SH-SY5Y/Bcl-xL (lanes 4, 5, and 6) non-treated cells were obtained and Bcl-xL expression was analysed by Western-blot. b) Densitometric analysis of Bcl-xL expression related to protein loading control α-tubulin. Data are expressed as mean ± S.E.M. of 3 experiments.
(TIF)

Figure S2 Presence of MCC channel in a mitochondrial inner membrane patch obtained from a SH-SY5Y/Bcl-xL cell. In this patch, an increase in Ca^2+^ concentration failed to induce MCC opening for 5 minutes prior to the beginning of the shown record. Pipette potential was changed to -60 mV at the beginning of the shown record. As it can be observed, the MCC was present and it was activated about 30 s after voltage change.
(TIF)

Video S1 SH-SY5Y/Neo cells were loaded with TMRM as indicated above and exposed to ionomycin (2 µM). Images were acquired using a fluorescence inverted microscope (Nikon Eclipse TE-2000-S, Birlingam, CA, USA) equipped with a 150 W Xenon lamp and 100×, 1.3 numerical aperture, epifluorescence oil immersion objective and commercial software (Metamorph, Universal Imaging Corporation, Silicon Valley, CA, USA). Frames were acquired every 15 seconds over 10 minutes. Fluorescence was observed using an excitation filter of 535 nm and an emission filter of 590 nm.
(AVI)

Video S2 Same as Video S1. but SH-SY5Y/Bcl-xL cells were used in the experiment.
(AVI)

Video S3 SH-SY5Y/Neo cells were loaded with calcein as indicated above and exposed to ionomycin (2 µM). Images were acquired using a fluorescence inverted microscope (Nikon Eclipse TE-2000-S, Birlingam, CA, USA) equipped with a 150 W Xenon lamp and 100×, 1.3 numerical aperture, epifluorescence oil immersion objective and commercial software (Metamorph, Universal Imaging Corporation, Silicon Valley, CA, USA). Frames were acquired every 20 seconds over 30 minutes. Fluorescence was observed using an excitation wavelength of 485 nm and an emission wavelength of 530 nm.
(AVI)

Video S4 Same as Video S3 but SH-SY5Y/Bcl-xL cells were used in the experiment.
(AVI)
