# Supplementary figures and images for: Bcl-xL Blocks a Mitochondrial Inner Membrane Channel and Prevents Ca2+ Overload-Mediated Cell Death
Source: PLoS One. 2011 Jun 2;6(6):e20423. doi: 10.1371/journal.pone.0020423 (PMC3107229; doi:10.1371/journal.pone.0020423)

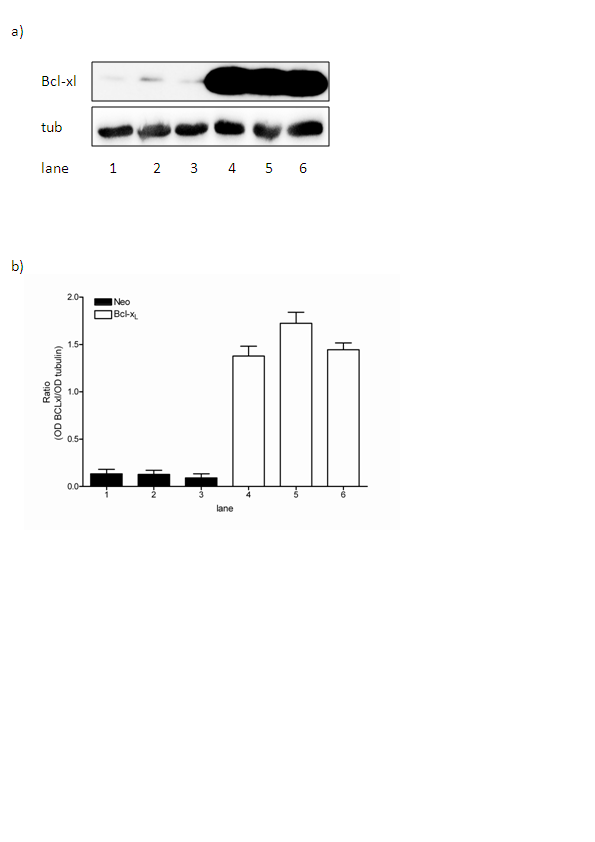

Supplement: Figure S1 — Bcl-xL expression in SH-SY5Y/Neo and SH-SY5Y/Bcl-xL cell. a) Total lysates obtained from SH-SY5Y/Neo (lanes 1, 2 and 3) and from SH-SY5Y/Bcl-xL (lanes 4, 5, and 6) non-treated cells were obtained and Bcl-xL expression was analysed by Western-blot. b) Densitometric analysis of Bcl-xL expression related to protein loading control α-tubulin. Data are expressed as mean ± S.E.M. of 3 experiments. (TIF) [file pone.0020423.s001.tif]

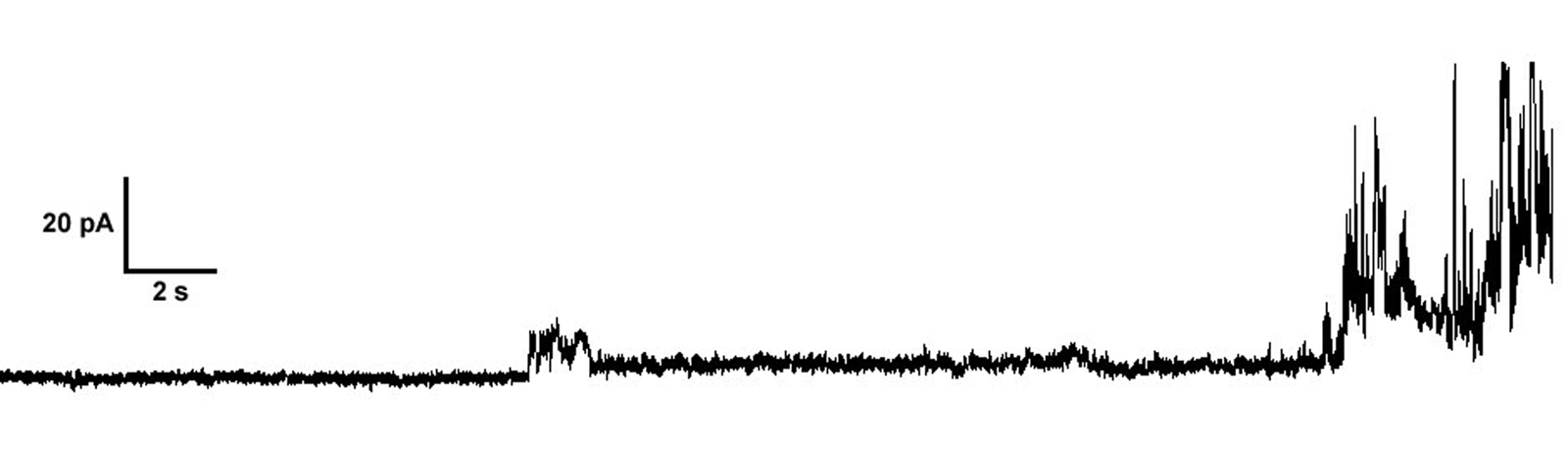

Supplement: Figure S2 — Presence of MCC channel in a mitochondrial inner membrane patch obtained from a SH-SY5Y/Bcl-xL cell. In this patch, an increase in Ca2+ concentration failed to induce MCC opening for 5 minutes prior to the beginning of the shown record. Pipette potential was changed to -60 mV at the beginning of the shown record. As it can be observed, the MCC was present and it was activated about 30 s after voltage change. (TIF) [file pone.0020423.s002.tif]
